# Thyroid Storm Superimposed on Gestational Hypertension: A Case Report and Review of Literature

**DOI:** 10.3390/medicina58030450

**Published:** 2022-03-20

**Authors:** Yen-Hua Chen, Chan-Pin Liao, Cheng-Wei Lu, Tzu-Yu Lin, Ya-Ying Chang

**Affiliations:** 1 Department of Anesthesiology, Far-Eastern Memorial Hospital, New Taipei City 220216, Taiwan; yanhua0624@gmail.com (Y.-H.C.); cpliao55@gmail.com (C.-P.L.); drluchengwei@gmail.com (C.-W.L.); drlin1971@gmail.com (T.-Y.L.); 2 Department of Mechanical Engineering, Yuan Ze University, Zhongli 320315, Taiwan; 3 International Program in Engineering for Bachelor, Yuan Ze University, Zhongli 320315, Taiwan

**Keywords:** caesarean section, hyperthyroidism, gestational hypertension, thyroid storm, cardiomyopathy, Burch–Wartofsky point scale

## Abstract

A thyroid storm is an extreme manifestation of thyrotoxicosis, and is life threatening without an early diagnosis. Pregnancy or childbirth may worsen maternal hyperthyroidism or induce the development of a thyroid storm. Gestational hypertension, a disorder defined as new-onset hypertension, develops after 20 weeks of gestation and shares symptoms with a thyroid storm. The diagnosis of a thyroid storm may be challenging in patients with gestational hypertension. To highlight the significance of early thyrotoxicosis-related gastrointestinal symptoms, we report a case of a 38-year-old woman with a twin pregnancy, who was diagnosed with gestational hypertension, and then developed a thyroid storm during the peripartum period. She complained of nausea and abdominal pain, followed by tachycardia, hypertension, and a disturbance of consciousness with desaturation. After emergency caesarean section, fever, diarrhea, and high-output heart failure, with pulmonary edema, were noted during the postoperative period in the intensive care unit. The diagnosis of a thyroid storm was confirmed using the Burch–Wartofsky point scale, which was 75 points. In this patient, the uncommon gastrointestinal symptoms, as initial manifestations of thyrotoxicosis, indicated the development of a thyroid storm. The distinguished presentation of thyrotoxicosis-induced cardiomyopathy and peripartum cardiomyopathy also helped in the differential diagnosis between a thyroid storm and gestational hypertension. Aggressive treatment for thyrotoxicosis should not be delayed because of a missed diagnosis.

## 1. Introduction

A thyroid storm is a rare and life-threatening condition, characterized by extreme manifestations of thyrotoxicosis, including hypertension, tachycardia, heart failure, and death [1]. In a previous report, the mortality rate of a thyroid storm was found to be as high as 30% in hospitalized populations [1]. The diagnosis of a thyroid storm is based on clinical features. The Burch–Wartofsky point scale (BWPS; Table 1) is widely used as a diagnostic tool for thyroid storms [2]. Moreover, thyroid function tests help quantify the levels of circulating thyroid hormones, including thyroxin (T4) and triiodothyronine (T3). The precipitating factors of a thyroid storm include surgery, trauma, infection, and poorly controlled hyperthyroidism [1]. In addition, pregnancy and childbirth can worsen hyperthyroidism [1]. During pregnancy, the thyroid gland increases in size by 10% to 40%, and the production of T4 and T3 increases by approximately 50% [3].

Gestational hypertension is a disorder that develops after 20 weeks of gestation, and is defined as a new-onset systolic blood pressure ≥140 mmHg, or as a diastolic blood pressure ≥90 mmHg, or both, on two occasions, at least 4 h apart. Severe gestational hypertension occurs when the systolic level reaches 160 mmHg, or the diastolic level reaches 110 mmHg, or both [4]. In clinical settings, severe gestational hypertension and thyroid storms share several manifestations. For example, patients with severe gestational hypertension and patients with a thyroid storm may both have profound hypertension. The similar clinical presentations may impede the early recognition of a thyroid storm in cases of gestational hypertension. Herein, we present a case of a thyroid storm in a pregnant woman with gestational hypertension. Our case highlights the distinct features of thyrotoxicosis, including uncommon gastrointestinal symptoms, which helped in the early diagnosis of the thyroid storm when superimposed on gestational hypertension. In this case report, we describe the treatment and anesthetic management of a thyroid crisis during the peripartum period, with emphasis on the differential diagnosis between a thyroid storm and gestational hypertension.

## 2. Case Report

A 38-year-old, gravida 2, para 1 (G2P1) woman with a twin pregnancy (164 cm/81 kg), at a gestational age of 35+5 weeks, was sent to the operating room for a caesarean section, due to a failure to progress to labor. The patient had no systemic diseases, except hyperthyroidism, for which she received treatment with propylthiouracil (PTU), at a dosage of 50 mg twice daily for 7 years. She maintained the treatment for hyperthyroidism throughout her pregnancy, and the free T4 level ranged from 1 ng/dL to 3 ng/dL. Six days before the caesarean section, the free T4 concentration was 2 ng/dL, which was within the normal range, presuming that the laboratory criteria for non-pregnant women were used. In addition, gestational hypertension was diagnosed at the gestational age of 24 weeks, during a prenatal examination. According to the medical records, her systolic blood pressure was gradually increased from 145 mmHg at 24 weeks of gestation to 175 mmHg at 35 weeks of gestation. Proteinuria was not observed. 

**Figure 1:**
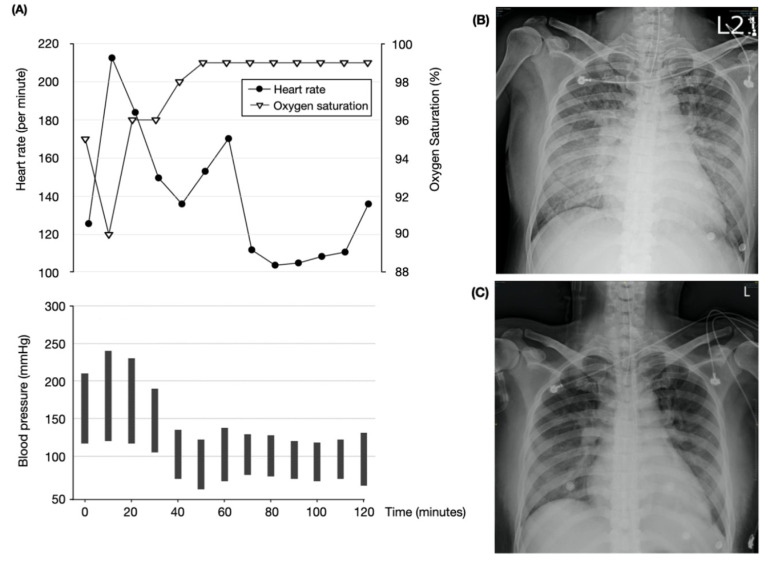
Intraoperative vital signs and postoperative chest radiography. (**A**) The patient had profound hypertension and tachycardia intraoperatively. (**B**) Chest radiography just after caesarean section showed bilateral pulmonary edema. (**C**) Pulmonary edema resolved after treatment on postoperative day 1. HR, heart rate; SpO_2_, oxygen saturation.

On arrival at the operating room, the patient was experiencing abdominal pain, accompanied by mild nausea. Tachycardia (heart rate of 124/min) and hypertension (210/117 mmHg), with an oxygen saturation of 95% on room air, were noted. During the preparation for spinal anesthesia, the patient complained of dyspnea. Thereafter, the oxygen saturation dropped to 90%, despite oxygen supplementation (10 L/min) using a simple mask. The arterial partial pressure of oxygen was 58 mmHg, and the electrolyte levels were within the normal range. The blood pressure quickly increased to 240/120 mmHg, and tachycardia, with a heart rate of 200/min, was noted. The patient became irritable, with a disturbance of consciousness. General anesthesia was immediately administered with propofol 100 mg and succinylcholine 100 mg, followed by tracheal intubation using a 7.0 mm endotracheal tube. Subsequently, the emergency caesarean section was performed, with two babies delivered (both had 1 min and 5 min Apgar scores of 7 and 9, respectively). The intraoperative vital signs are shown in Figure 1A. After the caesarean section, the patient was sent to the intensive care unit (ICU) for postoperative care.

In the ICU, fever, tachycardia, and diarrhea were noted. Chest radiography revealed bilateral pulmonary edema (Figure 1B). Echocardiography showed concentric hypertrophy of the left ventricle, and acute decompensated heart failure with a preserved ejection fraction. The postoperative free T4 level was higher than the preoperative level (Table 2). The diagnosis of a thyroid storm was confirmed using the BWPS, which was 75 points (based on manifestations including body temperature of 37.6 °C, agitation status, recent diarrhea, heart rate >140/min, pulmonary edema, and precipitant history). A continuous intravenous infusion of propranolol (2 mg per hour) and nicardipine (5 mg per hour) was administered for the treatment of hypertension and tachycardia. For the management of the thyroid storm, propylthiouracil (200 mg every four hours) and three doses of hydrocortisone (100 mg every eight hours) were administered. The hypertension and tachycardia resolved on postoperative day 1. Furthermore, improvements in the pulmonary edema were confirmed by chest radiography (Figure 1C), and the patient was extubated thereafter. The patient was discharged on postoperative day 5, without any further complications.

## 3. Discussion

Herein, we present a case of a thyroid storm in a gestational hypertensive pregnancy. The clinical presentations of a thyroid storm developed significantly before the caesarean section. The diagnosis of a thyroid storm was confirmed using the BWPS from manifestations including a body temperature of 37.6 °C, agitation status, recent diarrhea, heart rate >140/min, pulmonary edema, and precipitant history. Many cases of thyroid storms during pregnancy have been reported previously (Table 3) [1,5,6,7,8,9,10]. All of these cases developed a thyroid storm during the peripartum period, but none of these published case reports mentioned thyrotoxicosis-related gastrointestinal symptoms. It is noteworthy that our case manifested early gastrointestinal symptoms on arrival at the operating room. Previous reports suggest that gastrointestinal symptoms, such as hyperphagia and diarrhea, are commonly associated with thyrotoxicosis; whereas, vomiting and abdominal pain are uncommon [11]. Although some of these thyrotoxicosis-related gastrointestinal symptoms are unusual, they may be critical in the diagnosis of a thyroid storm. In our patient, gastrointestinal symptoms, including nausea and abdominal pain, developed before significant hemodynamic instability was noted. Of note, these gastrointestinal symptoms were the first signs of a thyroid storm in our case. We suggest that gastrointestinal discomfort must be carefully evaluated in pregnant women, especially in cases of hyperthyroidism during the peripartum period.

Similarly to our case, most patients had increased free T4 levels, by 2–4 ng/dL. According to the American Thyroid Association’s 2017 guidelines for the diagnosis and management of thyroid disease during pregnancy and the postpartum period, the interpretation of thyroid function tests may be affected by increased renal iodine excretion, increased thyroxine binding protein, and the thyroid stimulatory effects of human chorionic gonadotropin in pregnant women [3]. The free T4 levels continuously decline from the first trimester to the third trimester [12]. The free T4 level in the third trimester ranges from 0.54 ng/dL to 1.41 ng/dL, which is lower than the widely accepted reference range in non-pregnant women (0.9–2.3 ng/dL) [13]. In our patient, the level of preoperative T4 was within the reference range in non-pregnant women, but higher than the third trimester-specific reference range in pregnant women. A low TSH value in our case also indicated a status of hyperthyroidism before the caesarean section. Some reports indicate that the free T4 index is a reliable tool for the assessment of thyroid function during pregnancy [14,15]. Further clinical trials are needed to identify a practical marker to determine the level of thyroid hormones during pregnancy.

Both gestational hypertension and thyroid storms may lead to peripartum complications, including cardiomyopathy and poor fetal outcomes. Peripartum cardiomyopathy usually presents with dilated cardiomyopathy and heart failure, with either a reduced or preserved ejection fraction [16,17,18]. Although the mechanism of peripartum cardiomyopathy remains unknown, chronic inflammation, with a loss of myocytes, and fibrosis replacement have been suggested [16]. Thyroid storm-triggered cardiomyopathy usually presents with high-output heart failure and arrhythmia, such as atrial fibrillation [5,9,19]. Elevated levels of thyroid hormones reduce systemic vascular resistance and increase left ventricular contractility or blood volume, leading to an increase in cardiac output by 50% to 300% [19]. Our patient had high-output heart failure, without dilated chambers, which is a common presentation of thyrotoxic cardiomyopathy. Furthermore, fetal distress has been found in some cases of maternal thyroid storms [1]. Babies delivered by women with a thyroid storm may suffer from hyperthyroidism and require anti-thyroid therapy after birth [1]. Poor fetal outcomes, including fetal pulmonary complications, and even fetal death, have been reported (Table 3) [6,10]. The presence of a pediatrician may be helpful during an emergency caesarean section.

Rapid recognition of a thyroid storm in cases of gestational hypertension may be challenging. Differential diagnoses include preeclampsia with severe features, malignant neuroleptic syndrome, pheochromocytoma, and malignant hyperthermia. In our case, the diagnosis of a thyroid storm was confirmed based on several findings. Firstly, some thyrotoxicosis-related gastrointestinal symptoms in our patient, such as nausea, diarrhea, and abdominal pain, do not appear to be dominant in patients with gestational hypertension. Secondly, the BWPS score was high. Although we lack evidence supporting the good sensitivity or specificity of using BWPS scores in pregnant patients, the BWPS score is still a universal tool for the diagnosis of thyroid storms during pregnancy. Thirdly, our patient showed good responsiveness to postoperative antithyroid drug treatment. Rapid diagnosis and proper management led to good clinical outcomes. Maintaining a stable hemodynamic status remains the most important goal in the treatment of thyroid storms. The management of thyroid storms includes β-blocker (e.g., esmolol) infusion for a target heart rate less than 100/min, hydration to cool the body temperature, or medications, including thionamide or iodine solution [20]. The administration of corticosteroids also helps to increase vasomotor stability and decrease the conversion of T4 to T3 [21]. To prevent a peripartum thyroid crisis, keeping euthyroidism in pregnant patients can be achieved with PTU, instead of methimazole, which has teratogenic effects [22]. Furthermore, patients with a thyroid storm, who are scheduled for a caesarean section, may present challenges for anesthesiologists. The symptoms of dyspnea or thyrotoxicosis-related discomfort may make patients uncooperative or irritable during surgery. In addition, maternal pulmonary edema, with desaturation complicated by a thyroid storm, may lead to fetal hypoxia and acidosis. Therefore, we suggest the use of general anesthesia, instead of spinal anesthesia, for undergoing caesarean section in patients with a thyroid storm.

## 4. Conclusions

In summary, we reported a case of severe gestational hypertension in a patient who developed a thyroid storm immediately before undergoing a caesarean section. Early thyrotoxicosis-related gastrointestinal signs indicated the development of a thyroid storm. Aggressive treatment for thyrotoxicosis should not be delayed because of a missed diagnosis, due to the similar features between a thyroid storm and gestational hypertension.

## Figures and Tables

**Table 1:** The Burch–Wartofsky point scale. The Burch–Wartofsky point scale is widely used as a diagnostic tool for thyroid storms.

Temperature °F (°C)		Heart Rate (Beats/Min)	
99–99.9 (37.2–37.7)	5	<90	0
100–100.9 (37.8–38.2)	10	90–109	5
101–101.9 (38.3–38.8)	15	110–119	10
102–102.9 (38.9–39.2)	20	120–129	15
03–103.9 (39.3–39.9)	25	130–139	20
≥104.0 (≥40.0)	30	≥140	25
**Central Nervous System Effects**		Atrial fibrillation	10
Absent	0	**Congestive Heart Failure**	
Mild (agitation)	10	Absent	0
Moderate (delirium, psychosis)	20	Mild (pedal edema)	5
Severe (seizures, coma)	30	Moderate (bibasilar rales)	10
**Gastrointestinal-Hepatic Dysfunction**		Severe (pulmonary edema)	15
Absent	0	**Precipitating History**	
Moderate (diarrhea, nausea/vomiting, abdominal pain)	10	Negative	0
		Positive	10
Severe (unexplained jaundice)	20	-	
Total: <25 less likely, 25–45 impending thyroid storm, >45 thyroid storm			

**Table 2:** Levels of preoperative and postoperative free T4 and TSH. On postoperative day 1, the level of free T4 increased compared to preoperative level. Abbreviations: free T4, free thyroxine; TSH, thyroid-stimulating hormone.

	Preoperative Values	Postoperative Values	Reference Ranges
Free T4 (ng/dL)	2.00	2.46	0.90–2.30
3rd-generation TSH (μIU/mL)	0.038	0.049	0.400–4.000

**Table 3:** Cases of peripartum thyroid storm in previous case reports. The patient characteristics, delivery, clinical presentations, and prognosis are listed.

Reference	Patient Characteristic		Delivery	Presentation	Prognosis	
	Age (y)	History			Complication	Outcome
Sugiyama Y et al. (2017) [5]	36	Hyperthyroidism	CS	Fever, tachycardia, HTN, respiratory distress, agitation, bilateral pulmonary congestion, severe MR with LVEF >60%	Fetal hyperthyroidism	Residual MR
Kitazawa C et al. (2015) [7]	41	GA 36+6 weeks	NSD	Dyspnea, HTN, conscious disturbance, pulmonary edema, tachycardia, LVEF 48%		Discharge PPD 18
Yildizhan R et al. (2009) [10]	41	Graves’ disease. GA 27 weeks	NSD	Respiratory arrest (stridor), tachycardia	Fetal death	Patient discharge PPD 10
Okuda N et al. (2012) [6]	41	GA 32 weeks, PPROM	CS	HTN, tachycardia, desaturation, hepatic dysfunction and proteinuria, pulmonary infiltration, cardiomegaly, LV EF 43%	Baby incubated and sent to NICU.	Patient’s LVEF 60%
Ma Y et al. (2017) [1]	23	GA 36+4 weeks with PROM	CS	Fetal tachycardia, patient tachycardia, HTN, dyspnea, agitation, sweating, fever	Neonatal hyperthyroidism	Patient discharge POD 6
Peace JM et al. (2019) [8]	32	DM and Graves’ disease. GA 25 weeks with preterm labor.	CS	Tachycardia, profound thyroid function test, fever, HTN, agitated and dyspnea		Discharge POD 4
Lane AS et al. (2015) [9]	29	IDA, HTN. GA 29 weeks, placental abruption	CS	HTN, dyspnea, Af with RVR, LV and RV global hypokinesia, akinesia of the anterolateral wall of LV	Severe LV dilatation with global hypokinesia, severe MR	

Abbreviations: CS, caesarean section; GA, gestational age; HTN, hypertension; RV, right ventricle; MR, mitral regurgitation; LVEF, left ventricular ejection fraction; POD, postoperative day; NSD, normal spontaneous delivery; PPD, postpartum day; PPROM, preterm premature rupture of membrane; PROM, premature rupture of membrane; DM, diabetes mellitus; Af with RVR, atrial fibrillation with rapid ventricular response; IDA, iron deficiency anemia.

## Data Availability

Not applicable.
